# Complete and Incomplete Hepatitis B Virus Particles: Formation, Function, and Application

**DOI:** 10.3390/v9030056

**Published:** 2017-03-21

**Authors:** Jianming Hu, Kuancheng Liu

**Affiliations:** 1 Department of Microbiology and Immunology, Penn State University College of Medicine, Hershey, PA 17033, USA; kul21@psu.edu; 2 College of Life Sciences, Zhejiang Sci-Tech University, Hangzhou 310018, China

**Keywords:** hepatitis B virus, virion, empty virion, Australian antigen, HBsAg, HBcAg, subviral particles, CCC DNA, diagnosis, vaccine

## Abstract

Hepatitis B virus (HBV) is a para-retrovirus or retroid virus that contains a double-stranded DNA genome and replicates this DNA via reverse transcription of a RNA pregenome. Viral reverse transcription takes place within a capsid upon packaging of the RNA and the viral reverse transcriptase. A major characteristic of HBV replication is the selection of capsids containing the double-stranded DNA, but not those containing the RNA or the single-stranded DNA replication intermediate, for envelopment during virion secretion. The complete HBV virion particles thus contain an outer envelope, studded with viral envelope proteins, that encloses the capsid, which, in turn, encapsidates the double-stranded DNA genome. Furthermore, HBV morphogenesis is characterized by the release of subviral particles that are several orders of magnitude more abundant than the complete virions. One class of subviral particles are the classical surface antigen particles (Australian antigen) that contain only the viral envelope proteins, whereas the more recently discovered genome-free (empty) virions contain both the envelope and capsid but no genome. In addition, recent evidence suggests that low levels of RNA-containing particles may be released, after all. We will summarize what is currently known about how the complete and incomplete HBV particles are assembled. We will discuss briefly the functions of the subviral particles, which remain largely unknown. Finally, we will explore the utility of the subviral particles, particularly, the potential of empty virions and putative RNA virions as diagnostic markers and the potential of empty virons as a vaccine candidate.

## 1. Introduction

**Figure 1:**
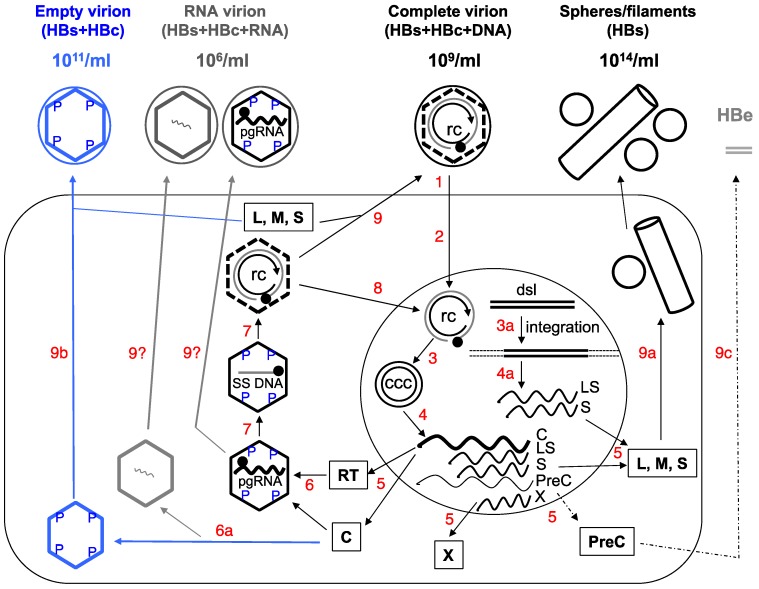
Schematic of hepatitis B virus (HBV) replication cycle. **1.** Virus binding and entry into the host cell (large rectangle). **2.** Intracellular trafficking and delivery of relaxed circular (RC) DNA to the nucleus (large circle). **3.** Conversion of RC DNA to CCC DNA, or integration of the double-stranded linear (DSL) DNA into host DNA (3a). **4. and 4a.** Transcription to synthesize viral RNAs (wavy lines), including the C mRNA for both the core and RT proteins; LS mRNA for the L envelope protein; S mRNA for the M and S envelope proteins; X mRNA for the X protein; and PreC mRNA for the PreCore protein. The C mRNA is also the pgRNA. **5.** Translation to synthesize viral proteins. **6.** Assembly of the pgRNA- (and RT-) containing NC, or alternatively, empty capsids (6a). **7.** Reverse transcription of pgRNA to synthesize the (−) strand SS DNA and then RC DNA. **8.** Nuclear recycling of progeny RC DNA to form more CCC DNA (intracellular CCC DNA amplification). **9.** Envelopment of the RC DNA-containing NC and secretion of complete virions, or alternatively, secretion of empty virions (9b) or HBsAg spheres and filaments (9a). Processing of the PreCore protein and secretion of HBeAg are depicted in 9c. The secretion of putative RNA virions is not yet resolved (9?). The different viral particles outside the cell are depicted schematically with their approximate concentrations in the blood of infected persons indicated: the complete, empty, or RNA virions as large circles (outer envelope) with an inner diamond shell (capsid), with or without RC DNA (unclosed, double concentric circle) or RNA (wavy line) inside the capsid respectively; HBsAg spheres and filament as small circles and a cylinder. It is important to point out that the concentrations of all these particles can vary widely between different patients and over time in the same patient. Intracellular capsids are depicted as diamonds, with either viral pgRNA, SS [(−) strand] DNA (straight line), RC DNA, or empty. The letters “P” denote phosphorylated residues on the immature NCs (containing SS DNA or pgRNA) or empty capsid. The dashed lines of the diamond in the RC DNA-containing mature NCs signify the destabilization of the mature NC, which is dephosphorylated. The empty capsids, like mature NCs, are also less stable compared to immature NCs but unlike mature NCs, are phosphorylated. The soluble, dimeric HBeAg is depicted as grey double bars. The thin dashed line and arrow denote the fact that HBeAg is frequently decreased or lost late in infection. Boxed letters denote the viral proteins translated from the mRNAs. The filled circle on RC DNA denotes the RT protein attached to the 5’ end of the (−) strand (outer circle) of RC DNA and the arrow denotes the 3’ end of the (+) strand (inner circle) of RC DNA. ccc, CCC DNA; rc, RC DNA. For simplicity, synthesis of the minor DSL form of the genomic DNA in the mature NC, its secretion in virions, and infection of DSL DNA-containing virions are not depicted here, as are the functions of X. See text for details. Modified from [2].

A major characteristic of hepatitis B virus (HBV) is the secretion of large amounts of complete and incomplete viral particles. Assembly of the complete HBV virions, which are spheres 42 nm in diameter and routinely found in the blood of infected patients at 10^9^/mL, begins with the packaging of the viral pregenomic RNA (pgRNA), as a complex with the viral reverse transcriptase (RT) protein, into an icosahedral capsid, 30 nm in diameter and composed of 240 copies of the viral capsid or core protein (HBc; also called HBV core antigen or HBcAg) (Figure 1). Within the capsid, RT first converts pgRNA to a single-stranded (SS) (minus-sense) DNA, and subsequently to a partially double-stranded (DS), relaxed circular DNA or RC DNA [1]. Somewhat used as a jargon, the capsid together with its interior RNA or DNA content is called a nucleocapsid or NC. NCs with pgRNA and SS DNA are considered immature as they are incompetent for envelopment and secretion as virions. In contrast, RC DNA-containing NCs are considered mature and are selected for envelopment by the viral envelope or surface proteins (also called HBV surface antigen or HBsAg) and secreted extracellularly as complete virions [2,3,4,5,6]. As a result, a complete HBV virion contains an outer envelope enclosing an inner capsid, which in turn encloses the RC DNA genome (Figure 1).

In contrast, incomplete or subviral particles of HBV, of which there are two known major types, are either missing both the genome and capsid or missing just the genome (Figure 1). The first type is the classical HBsAg spheres and filaments of 20 nm in diameter (HBsAg particles), historically also called Australian or Au antigen owing to its initial identification in an Australian aborigine. These subviral particles are composed of only the viral surface proteins and found in the blood of infected individuals at up to 100,000-fold in excess over the complete virions (at 10^14^/mL) [7]. The second class is the recently discovered empty (genome-free) virions, which contain the surface proteins enclosing the viral capsid but no genome and are typically found at 100-fold higher levels over complete virions in the blood of infected individuals (10^11^/mL) [8,9]. In addition, putative particles containing HBV RNA, at much lower levels than the other particles (100- to 1000-fold lower than complete virions) have also been reported recently (Figure 1) [10,11,12,13]. 

HBV-infected cells also secrete a soluble, dimeric protein antigen called hepatitis B e antigen (HBeAg) (Figure 1) [14]. HBeAg is processed from the so-called PreCore (PreC) protein and shares most of its amino acid residues with HBcAg but has an N-terminal extension of 10 amino acid residues (unique to the PreC region) and a C-terminal truncation of ca. 34 amino acid residues, relative to HBcAg. Independent of capsid assembly or viral replication, HBeAg is released through the host cell secretary pathway and may exert immunoregulatory effects [15]. Historically, serum HBeAg has been widely used to monitor viral infection and treatment response as it is usually associated with high levels of viral replication [15]. In line with the focus on HBV particles here, we will not discuss this soluble viral antigen anymore. 

Secretion of all HBV particles and proteins, as well as viral replication, are ultimately directed by a viral episome, the covalently closed circular DNA (CCC DNA). CCC DNA is synthesized from RC DNA, which is released either from complete virions infecting from outside the cell or from intracellular mature NCs (via the so-called intracellular CCC DNA amplification pathway), in the nuclei of infected hepatocytes and serves as the sole viral transcriptional template able to direct the expression of all viral gene products and sustain viral replication (Figure 1) [1,16,17,18]. Ongoing efforts to develop a cure for chronic HBV infection ultimately are aimed at the elimination of CCC DNA (or stable silencing of its transcriptional activity). It is thought that CCC DNA is very stable and may persist in the absence of viral replication for as long as the infected cell survives [19,20,21]. Alternatively or additionally, new CCC DNA is likely being continuously produced in the infected liver, even during apparently effective antiviral therapy, from residual replicative viral DNA via the afore-mentioned intracellular amplification pathway whereby the end product of reverse transcription, RC DNA, is directed to the host cell nucleus to form more CCC DNA (instead of being secreted in virions), and perhaps also via de novo infection by residual complete virions. This is possible because inhibition of RC DNA synthesis is incomplete with current antiviral therapy, which targets only the DNA synthesis activity of RT [22,23]. Nevertheless, current treatment can lead to significant reduction or loss of CCC DNA in a minority of patients via a mechanism(s) that is yet to be elucidated but likely involves a combination of reduced CCC DNA formation (due to the depletion of RC DNA), loss of infected cells, and perhaps degradation of preexisting CCC DNA. 

It has proven challenging to monitor intrahepatic CCC DNA level or its transcriptional activity either directly using infected liver tissues, or indirectly by measuring surrogate markers in the periphery (blood or serum) [2,24,25]. Detection of CCC DNA in liver biopsies may be the most direct way for this purpose. However, it is impractical to perform this invasive and risky procedure on a routine basis due to safety concerns. Also, as infection of the liver is likely to become increasingly heterogeneous during long-term infection and antiviral treatment due to loss or decrease of CCC DNA in some but not all infected hepatocytes, it is unclear whether liver biopsies, which can obtain only very small amounts of tissues, can faithfully reflect the infection status throughout the liver. Additionally, it remains technically challenging to specifically and accurately detect CCC DNA in clinical samples, which are usually contaminated with a large excess of RC DNA that is structurally related to CCC DNA (Figure 1) and difficult to discriminate from CCC DNA in PCR-based methods that are needed to detect the small amounts of CCC DNA [18]. As will be detailed later in this article, the various HBV particles released into the blood stream of infected patients have attracted increasing attention in recent years as convenient and accessible surrogate markers to monitor intrahepatic CCC DNA level and transcriptional activity. 

Below, we will first summarize current understanding of the molecular processes that lead to the release of HBV virions and subviral particles. The potential role of these particles in viral replication and pathogenesis will also be briefly discussed. The emphasis will be on the recently discovered empty virions and potential RNA virions. We will then focus on the possible application of the various viral particles as convenient peripheral biomarkers to monitor viral activity in the infected liver, and the potential of empty virions as a candidate for a new generation of HBV vaccine. 

## 2. Complete and Incomplete (Subviral) HBV Particles Secreted during HBV Replication

### 2.1. HBsAg Spheres and Filaments (Au Antigen) 

The abundance of the classical HBsAg spheres and filaments in the blood of HBV infected patients greatly facilitated the discovery of HBV even before the characterization of its genetic materials [2,7]. There are three HBV envelope proteins, called large (L), middle (M), and small (S) (Figure 1), among which S is the predominant protein present in both virions and HBsAg particles and L is enriched relatively in virions and filaments and barely detectable in spheres [26]. In accordance with their relative abundance of L, virions and filaments appear to share a similar pathway of secretion involving the host cell endosomal sorting complexes required for transport (ESCRT) components and the multivesicular bodies (MVBs), whereas spheres are thought to be secreted via the constitutive secretory pathway of the host cell [27,28]. 

### 2.2. Complete Virions 

A defining feature of HBV morphogenesis, as a member of the *Hepadnaviridae*—a group of para-retroviruses that replicate a DNA genome via reverse transcription of a RNA intermediate, is the strict selectivity for mature NCs containing RC DNA for envelopment and secretion [6]. Thus, complete HBV virions contain a DS DNA genome, in contrast to conventional retroviruses that contain a SS RNA genome. To accomplish this selective virion formation, only the “correct” NCs, i.e., mature ones containing RC DNA, but not immature ones containing SS DNA or pgRNA, are selected for envelopment during virion formation (Figure 1) [3,4,5,6,8,29]. Mature NCs are thought to acquire the host-derived lipid bilayer studded with the viral envelope proteins via budding into the lumen of an intracellular membrane thought to represent MVBs [27,28,30] for extracellular secretion. As mentioned earlier, complete virions are known to be enriched (relative to HBsAg spheres) for the L protein, which along the S protein (but not the M protein), are required for envelopment of mature NCs and secretion of complete virions [31]. Specifically, the C-terminal portion of the PreS1 region of L was identified as a “matrix” domain (MD) that is thought to recognize mature NCs [32,33]. 

**Figure 2:**
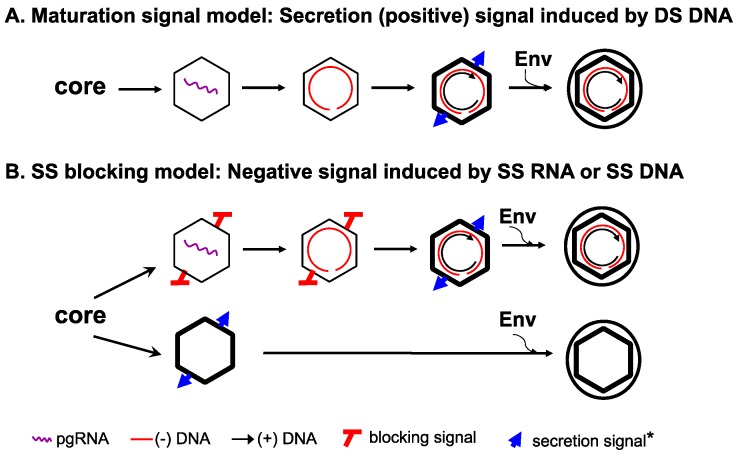
The single strand blocking hypothesis to explain selective HBV virion formation. The new hypothesis is presented in panel (**B**), in comparison with the classical maturation signal hypothesis depicted in panel (**A**). The symbol * denotes that the envelope signal for the mature NC vs. the empty capsid may or may not be the same. See text for details. Modified from [8].

How genome maturation, within NCs, is coupled to envelopment, from without, remains poorly understood. In particular, the exact nature of the viral genome that is ultimately responsible for regulating virion secretion is not yet clear. As SS RNA or DNA is not secreted in virions but DS DNA, as RC DNA or a minor, double-stranded linear (DSL) form, or RNA-DNA hybrid, is [3,4,5,29,34,35], it was hypothesized that the accumulation of DS DNA as a result of second strand elongation during reverse transcription triggers a structural change in the maturing NC that, in turn, signals envelopment and secretion [4,6,36,37]. Thus, this so-called maturation signal would emerge on the mature NC only as reverse transcription approaches completion and positively regulate virion secretion (Figure 2A). 

Regardless of the exact nature of the secretion signal, the HBc protein likely plays an integral role in the selection of mature NCs for virion formation as it forms the NC shell and is thus situated appropriately to transmit the nature of the nucleic acid inside the NC (RC DNA vs. SS DNA or pgRNA) to its exterior so as to allow the viral envelope proteins to differentiate NCs with different maturity. In other words, the envelope proteins are thought to sense, indirectly, the interior content of the maturing NC through maturation-associated structural changes on the capsid surface, which could constitute the elusive maturation signal. Indeed, mutations of specific residues located on the capsid surface, genetically defined as the so-called matrix binding domain (MBD), can block virion formation while still allowing RC DNA synthesis [38,39,40]. It is not yet clear if and how these residues might be involved in either sensing the interior nucleic acid content of the NC or mediate the interactions between the mature NC and the viral envelope proteins. On the other hand, the destabilization of mature NCs, relative to immature NCs [41], may provide a structural property for the envelope proteins to distinguish the mature from immature NCs (Figure 1). In addition, NC dephosphorylation has been shown to associate with, and be required for, maturation but it is not required for NC envelopment *per se* (Figure 1) [36,42,43]. 

### 2.3. Empty (Genome-Free) Virions (Enveloped Capsids)

Given the above discussion on selective HBV virion morphogenesis, it came indeed as a great surprise that genome-free (empty) HBV virions are also secreted, in even larger amounts than the RC DNA-containing complete virions (Figure 1 and Figure 2). In retrospect, empty virions had probably been sighted even before the discovery of HBV reverse transcription. Four decades ago, it was reported that two populations of HBV virion particles circulated in the blood of infected patients, with one having a lighter buoyant density than the other [44,45,46]. These so-called “light” virion particles contained HBV envelope and core proteins but in contrast to the “heavy” particles, displayed no endogenous polymerase activity, which reflects DNA synthesis by the virion RT protein using the endogenous RC DNA template. These light particles also appeared empty under electron microscopy and were assumed to be devoid of viral DNA. While these early reports hinted at the presence of empty virions, they did not directly determine the levels of viral DNA, if any, in the light virions or whether they contained viral RNA or host nucleic acid. It had also remained possible that the light particles simply represented damaged virions from which the RC DNA genome had leaked out. A more recent study suggested that the light virion particles might actually contain, instead of the normal capsid protein, an aberrantly processed PreCore protein related to the aforementioned HBeAg [47] (see below also). Another recent report found that small amounts of enveloped HBV capsids devoid of viral genome were secreted in transfected cell cultures but those were again deemed aberrant [35]. Thus, it had remained unclear if HBV secretes DNA-free virions and if so, whether it is part of the normal virion morphogenesis process. 

We first stumbled upon empty HBV virions when we noticed that apparently enveloped capsids continued to be secreted into the culture supernatant unabated, despite profound inhibition of HBV DNA synthesis and thus loss of DNA-containing virions when the antiviral protein, Apobec3G (apolipoprotein B mRNA editing enzyme catalytic subunit 3G), was co-expressed in hepatoma cells in which HBV was replicating [8,48]. In addition, we noticed that the peaks in CsCl density gradients of viral DNA and capsid in HBV virions, harvested from cell culture supernatant, or from sera of infected chimpanzees or patients, never coincided; rather, the virion capsid peak was always just above (i.e., with lighter density than) the virion DNA peak [8,9]. Third, a rough estimate of the amount of viral DNA vs. that of the capsids in the virions indicated that there were many more capsids for the amount of DNA; indeed, more than 99% of virions contained no DNA [8,9]. Finally, enveloped capsids (i.e., empty virions) are secreted abundantly in the complete absence of either pgRNA packaging or viral DNA synthesis [8] as shown by the use of viral mutants unable to support these processes. 

To reconcile the apparent stringency in selecting mature (but not immature) NCs for complete virion formation and the secretion of empty virions containing no genome at all, we proposed a SS DNA (or pgRNA)-dependent “blocking signal” that is induced in immature NCs, which actively prevents their envelopment (Figure 2B) [8]. The empty capsids, being devoid of any nucleic acid, lack such a negative signal and can thus, by default, be enveloped and secreted as empty virions (Figure 2B). According to this model, the apparent requirement of RC (DS) DNA for envelopment of mature NCs and secretion of DNA-containing virions would NOT be due to the need for the DS DNA per se, as envisioned by the classical maturation signal model (Figure 2A) [4,6,36]. Rather, the requirement for RC DNA synthesis in complete virion secretion reflects the need to remove or sequester the pgRNA or SS DNA from NCs, the trigger of the blocking signal, so as to relieve its inhibition on NC envelopment. Structurally, empty capsids, like mature NCs, are less stable than immature NCs [41], which may be a shared property that allow their recognition and selection by the envelope proteins for virion formation (Figure 1). Alternatively, the secretion of empty virions could be explained, in principle, by invoking a second envelopment signal for empty capsids, distinct from that emerging on RC DNA-containing mature NCs (Figure 2). Detailed analyses of the requirements from the capsid and the envelope proteins for empty virion secretion, in comparison with those for secretion of complete virions, should help define the capsid-envelope interactions needed for the secretion of empty and complete virions. 

The secretion of empty HBV virions extracellularly requires assembly of empty capsids inside cells (Figure 1). Indeed, empty HBV capsids are assembled at high levels in human cells in culture [8,49], in insect cells [50,51], and likely also in the HBV-infected liver [45,46,52]. Thus, in contrast to capsids assembled in bacteria that package non-specific RNAs [53,54], HBV capsids assembled in authentic host cells do not package non-specific RNAs and are empty if they fail to package the viral pgRNA. This is likely due to the extensive capsid phosphorylation in human and insect cells (but not in bacteria), which decreases its RNA binding activity and prevents non-specific RNA packaging [49,51,55,56].

#### Do Empty Virions Contain an Aberrant Core Protein, HBcrAg? 

Over 10 years ago, there was a report claiming that an aberrant protein derived from the aforementioned PreCore protein, dubbed HBc related antigen or HBcrAg, in some DNA-free HBV virions in patient sera [47]. The protein migrated as a 22 kd protein (hence p22cr) on sodium dodecyl sulfate-acrylamide gels. Western blot analysis using HBc/HBe specific antibodies and mass spectrometry appeared to identify HBcrAg as an aberrantly processed PreCore protein containing the entire PreC region including the uncleaved signal peptide (i.e., with a 29 residue N-terminal extension relative to HBc or 19 residue extension relative to HBe) but similar to HBe, lacking the C-terminal arginine-rich domain or CTD (ending at ca. 150), which mediates RNA and DNA binding by HBc. It remains unclear how this aberrant PreCore protein could assemble into capsids as the PreC region is known to disrupt capsid assembly via an intramolecular disulfide bond involving a Cys residue in the PreC region [14], or if the presumed capsid is made, how it would be enveloped and secreted. No follow-up studies have been reported by the authors or others to confirm this report. On the other hand, we have shown that empty virions clearly contain capsids consisting of the normal HBc protein and no aberrant core related protein or any PreC region sequence is needed for empty virion secretion [8]. Furthermore, using multiple antibodies specific for the HBc CTD, which was supposedly missing from HBcrAg in empty virions, including one antibody whose epitope includes the last Cys (183) residue [49], we could detect the normal HBc protein in empty virions in cell culture supernatant and in human serum samples, indicating clearly they contain the entire CTD [43]. At this point, it seems likely that the so-called HBcrAg might have been an artifact of detection and in any case, it is certainly not required for empty virion secretion.

### 2.4. RNA Virions

Even more surprising than the discovery of empty HBV virions, it appears that HBV may also secrete, albeit at a low level, RNA-containing particles (Figure 1), thus blurring the distinction between para-retroviruses and conventional retroviruses. Apparently inspired by the need to identify easily accessible surrogate markers for monitoring hepatic CCC DNA, there have been a flurry of reports recently on the detection of HBV RNA in serum samples of HBV infected patients [10,11,12,13,57,58]. Most of these suggest that HBV RNA levels detected in the patient sera are around 10^6^ copies/mL, ca. 0.1%–1% of HBV DNA levels, in the absence of antiviral treatment [10,11,13,57,59,60].

The nature of the viral RNA as well as the physical entity that the RNA is associated with in the blood remain to be better characterized. Some evidence has been reported suggesting that the HBV RNA in serum may be associated with viral capsids as demonstrated by anti-HBc immunoprecipitation [57]. The fact that detergent treatment enhanced the immunoprecipitation efficiency was consistent with a possible association of the RNA with enveloped virions [57], although other membranous vesicles were not excluded. It remains possible that at least some HBV RNA detected in the serum samples is released in vesicles, e.g., as a result of lysis of infected hepatocytes, independent of viral capsid or virion formation. Consistent with this possibility is the detection of truncated HBV RNAs, perhaps as fusion to cellular RNAs and transcribed from integrated HBV DNA, were detected in the blood, in the absence of any other HBV markers [11,58]. 

The nature of the HBV RNA in the serum similarly remains to be clarified. Some reports suggest that the RNA could be authentic pgRNA [10,57]. If pgRNA is indeed secreted in virions, it may reflect a less than complete control in the selection of mature NCs for envelopment, leading to envelopment of some pgRNA-containing immature NCs (Figure 1). In this scenario, some HBV virions would be secreted containing a RNA genome, similar to a conventional retrovirus, and would also be expected to harbor the viral RT protein that is essential for pgRNA packaging [2]. If this indeed occurs, it would be interesting to test if these HBV “RNA virions” are infectious. In this regard, it is important to note that secretion of “immature” virions containing SS DNA has been demonstrated by certain HBc mutants [61]. Furthermore, the snow goose hepatitis B virus (SGHBV) naturally secretes SS DNA-containing virions [62], in contrast to all other hepadnaviruses identified to date, and two specific residues (74 and 107) in the SGHBV core protein have been identified as responsible for this immature secretion phenotype [63]. Therefore, secretion of immature virions containing SS DNA can clearly occur in certain cases, suggesting that RNA virion secretion can, in principle, also occur under certain conditions.

On the other hand, it remains possible that the HBV RNA detected in the serum may represent residual pgRNA sequences present in minus strand DNA-pgRNA hybrids, which are known to be secreted (likely by mimicking DS DNA) [3,34] and whose levels in the blood can be enhanced when viral DNA synthesis is blocked by a RT inhibitor [64]. In addition, a small amount of “empty virions” may not be completely empty. Thus, while most capsids assembled in infected hepatocytes would be empty if they fail to package pgRNA as discussed above, a small fraction of capsids may package small amounts of HBV or host RNA nonspecifically (and without packaging the RT protein) (Figure 1). These non-specifically packaged RNAs may not be sufficient to trigger the blocking signal that is postulated to be induced by pgRNA (see above) to negatively regulate NC envelopment and virion secretion. In particular, our recent studies have shown that a decrease or block of HBc CTD phosphorylation can lead to packaging of non-specific RNA (mostly tRNA-sized small RNAs) by HBV capsids in human cells (Figure 1), analogous to non-specific RNA packaging by (non-phosphorylated) HBV capsids in bacteria [49]. 

It is important to point out that certain technical uncertainties have likely confounded the interpretation of some conflicting results on possible RNA virions in the literature. For example, some authors using hepatoma cell culture supernatant as a source of HBV virions, without, seemingly, attention to the fact that naked capsids (without the envelope) containing pgRNA, SS DNA or DS DNA, are routinely released to such supernatant (e.g., see [8]). Also, since the DNA-containing virions are usually present at high levels, routine DNase digestion, meant to remove the viral DNA before RNA detection, might not have been sufficient to completely remove all viral DNA such that residual HBV DNA, rather than, or in addition to, RNA, was actually being measured in some cases. 

## 3. Functional Significance of the Subviral Particles

Whereas the major function of complete virions is obviously to deliver the viral genome to a newly infected cell, why HBV releases such an excessive amount of subviral particles remains a fascinating but challenging question still today. In the case of HBsAg particles, it is certainly reasonable to argue that a major role of these particles for the virus is to sequester the host antibodies against the HBsAg so as to block the neutralizing effect of these antibodies against complete virions. Beyond that, additional immune modulatory functions of HBsAg particles, if any, remain to be resolved [65]. Similarly, the potential functions of empty virions remain to be defined. As the spatial arrangement of the viral envelope proteins on the surface of empty virions likely mimic that in complete virions better than that in the HBsAg particles, empty virions may be more potent in sequestering virion-targeted HBs antibodies. Furthermore, the internal capsid in the empty virion may play some additional role that is completely distinct from the envelope proteins. For example, empty virions, like complete virions, can probably enter cells to deliver the capsid intracellularly, which may, in turn, play some intracellular role during the initial stage of infection before new HBc proteins are made. In this regard, some suggestion has been made for a role of HBc in regulating CCC DNA transcription or host innate immunity [65,66], which could involve the capsids from the incoming virions (predominantly from empty virions). As for the putative RNA virions, it remains unclear if they would play any roles beyond those by HBsAg particles and empty virions. In principle, the putative virion RNA could be delivered to an infected cell to exert some function early during infection before new viral RNA is made, analogous to what is proposed above the capsid in empty virions. However, the low abundance of RNA virions (less than 1% of complete virions, if they are indeed secreted) seems to make this unlikely. 

## 4. Potential Applications of HBV Particles in Clinical Management

Historically, the abundance of subviral particles has proven very useful in both the diagnosis and management of HBV infections. As mentioned above, the classical Au antigen (i.e., HBsAg) heralded the identification of the virus itself [7]. Furthermore, since its discovery, the HBsAg particles have been used extensively as a convenient marker to monitor HBV infection, screening of blood supplies, and gauge response to antiviral treatment (see more below). Similarly, the Au antigen particles, harvested directly from pooled plasma of infected patients, formed the basis of the 1st generation of HBV vaccine, which has proven to be very safe and highly effective [7]. As will be discussed below, empty virions, and possibly RNA virions, may also prove to be valuable as diagnostic markers and perhaps, as a new vaccine candidate. 

### 4.1. Diagnostics

The long-standing and wide-spread use of complete virions (clinically measured as viremia or viral DNA load in the blood) and HBsAg particles for diagnostic purposes have been reviewed recently [24,25,67,68], and will not be discussed in detail here. It is important to emphasize, however, that recent efforts to use quantitative measurements of serum HBsAg, and the soluble HBeAg, as surrogate markers to monitor the intrahepatic CCC DNA, the basis of HBV infection and persistence, have revealed each of these has major limitations. In the case of HBsAg, it is well known that it can be expressed from integrated HBV DNA (Figure 1), which accumulates over time (by at least 10- to 100-fold) [68,69,70,71,72] and could be the predominant source of serum HBsAg (instead of CCC DNA). Integrated HBV DNA is derived predominantly from the minor form of viral DNA, DSL (Figure 1) [69,73] in which the PreCore/Core gene is disrupted but the HBsAg gene is maintained [74,75]. This likely explains the loss of correlation between serum HBsAg levels and hepatic CCC DNA, especially in the late stage of HBV infection [76,77,78]. Another issue that can affect the accuracy of HBsAg quantification is the frequent mutations in the envelope gene, some of which are induced by current antiviral treatment targeted at the RT protein whose coding sequences overlap with the envelope gene (Figure 1) [68,79]. On the other hand, expression of HBeAg (like that of HBcAg) is unlikely to be directed from integrated HBV DNA, due to the disruption of the PreCore/core gene, and could, in principle, be a good surrogate for hepatic CCC DNA (Figure 1). However, HBeAg can be decreased or lost entirely due to frequent mutations in the core promoter or the PreC region, as discussed recently [2,9]. In addition, viremia (HBV DNA or complete virions in the blood) is routinely suppressed to very low or undetectable levels (despite the persistence of CCC DNA in the liver) following current antiviral therapy, rendering complete virions useless as a marker to monitor CCC DNA persistence in this situation. Thus, there is increasing realization that better markers are needed to monitor HBV infection, particularly the hepatic CCC DNA. As discussed below, empty virions, and perhaps RNA virions, have the potential to serve as better surrogate markers for hepatic CCC DNA. 

#### 4.1.1. Serum Empty Virions (HBcAg) as a Marker for Hepatic CCC DNA

Since the production of empty virions is uncoupled from viral DNA replication but requires the expression of both HBcAg and HBsAg, they can in principle serve as an effective biomarker for transcriptionally active CCC DNA during current antiviral therapy with RT inhibitors, which, as mentioned above, can reduce serum HBV DNA (i.e., complete virions) to undetectable levels but has no direct effect on CCC DNA level or its transcriptional activity [78,80]. As mentioned above, a minority of the treated patients do experience significant decreases in hepatic CCC DNA with current antiviral therapy, presumably due to host-mediated elimination of infected cells or CCC DNA and the therapy-induced elimination of the CCC DNA precursor (i.e., RC DNA) and consequently, diminished replenishment of the CCC DNA pool [78,81,82,83]. In a pilot study to evaluate the potential of serum empty virions as a surrogate biomarker for hepatic CCC DNA, we determined serum levels of empty virions, using a relatively insensitive western blot assay (with a detection limit of ca. 50 ng/mL), together with viral DNA (complete virions) and HBsAg levels, in a small group of patients who underwent treatment with the RT inhibitor tenofovir [9]. Levels of serum empty virions can be monitored conveniently by measuring serum HBcAg levels, as the amount of HBcAg in complete virions contributes to less than 1% of the total serum HBcAg (i.e., from both complete and empty virions).

Before tenofovir treatment, serum empty virions were present at levels up to 10^11^/mL and at more than 50- to 100,000-fold excess compared to RC DNA-containing virions [9], consistent with our earlier observations in HBV-infected chimpanzees and in the supernatants of HBV-replicating hepatoma cells [8]. Following the antiviral therapy, the secretion of complete virions decreased dramatically (by ca. 10^7^/mL or more) in virtually all patients, as expected. However, secretion of empty virions was not decreased in most cases even after years of potent HBV DNA suppression. This is, of course, exactly as predicted given that DNA synthesis is required for secretion of complete virions, but completely dispensable for empty virion secretion, which would continue unabated unless the hepatic CCC DNA is eliminated or stably silenced. Significant reductions in, and possibly complete loss of, serum empty virions were in fact observed in a minority of treated patients, including those who achieved HBsAg loss. The results suggest that these patients might indeed have experienced significant reductions or loss in hepatic CCC DNA levels (or CCC DNA transcriptional activity) after the therapy, although the unavailability of the corresponding liver tissues unfortunately precluded a direct measurement of hepatic CCC DNA in these patients. Future studies with larger sample sizes and more sensitive HBcAg assay format, together with direct measurements of corresponding hepatic CCC DNA levels, will be needed to further assess the usefulness of serum empty virions as a surrogate marker for hepatic CCC DNA and to determine the potential diagnostic and prognostic significance of serum empty virions. As the HBc sequence is known to be more variable in some regions than in the others [84], epitopes will have to be carefully considered for antibody-based assays to detect serum HBcAg (i.e., empty virions). 

Interestingly, attempts were made over two-decade ago to measure serum HBc, before the realization of empty virion secretion. An enzyme-linked immunosorbent assay (ELISA) kit was developed to measure serum HBcAg, using an antibody specific for the HBc CTD, which indeed showed a good correlation between serum HBc and HBV DNA in untreated patients, and furthermore, the decrease of serum HBc was much less than that of serum HBV DNA during treatment with an RT inhibitor (lamivudine in this case) in the single patient who was monitored [85]. These results led the authors to speculate the secretion of DNA-free virions although they didn't provide any other supporting evidence. In the meantime, a different ELISA kit was developed to measure the so-called HBcrAg in serum samples, which has been used in a number of clinical studies as a putative surrogate in attempt to monitor hepatic CCC DNA. 

It is important to point out that some confusions exist in the literature as to what “HBcrAg” is exactly. Initially, in 2002, an ELISA kit was reported for the detection of both the soluble HBeAg and HBcAg that was released from virions in serum samples, using antibodies targeted to the sequences shared by both HBc and HBe [86]. A few years later, an aberrant HBeAg and HBcAg related protein, the so-called p22cr (with an even longer N-terminal extension than HBe and missing the CTD of HBc; see above), was claimed to be present in DNA-free HBV virions in serum samples from HBV infected individuals. This aberrant protein was also named HBcrAg. In the literature, the term “HBcrAg” has been used to describe a combination of HBc and HBe [13,86,87,88]; the supposedly aberrant p22cr [47]; and a combination of all three entities, HBc, HBe, and p22cr [89,90,91]. As discussed above, the so-called p22cr probably does not really exist and the current HBcrAg ELISA kit most likely detects a combination of HBcAg (released from virions) and HBeAg. Thus, with HBeAg present, the HBcrAg kit detects both serum empty virions and HBeAg but in the absence of HBeAg, it essentially detects empty virions (again with the contribution of serum HBcAg from complete virions being negligible). 

Not surprisingly, serum HBcrAg levels were found to correlate relatively well with serum HBV DNA and HBsAg levels, to decrease in HBeAg (−) phase, and to decrease much slower than serum HBV DNA and remains detectable in serum HBV DNA (−) patients upon nucleoside reverse transcriptase inhibitor (NRTI) treatment [13,88,90], as we observed for serum empty virions [9]. Furthermore, serum HBcrAg levels were correlated with CCC DNA levels and frequency of HBcAg (+) hepatocytes in the liver and with liver disease activity [87,89,92,93]. It was also found to predict liver cancer development and recurrence in HBV-infected patients, and was an even better predictor than serum HBV DNA [92,94]. Serum HBcrAg was also found to associate with reactivation of occult hepatitis B following immunosuppressive therapy [91].

As discussed above, serum HBeAg levels may not strictly correlate with hepatic CCC DNA levels or its transcriptional activity, due to the frequent mutations in the PreC region and core promoter. Therefore, measurement of both serum HBeAg and HBcAg, by using the HBcrAg assay, may not entirely reflect functional CCC DNA in the liver, since CCC DNA defective in HBeAg expression can nevertheless still express HBcAg and the other viral proteins and sustain HBV persistence. Given the aberrant “p22cr” or “HBcrAg” is most likely an artifact, as discussed above, it will be advantageous to measure exclusively serum HBcAg (i.e., empty virions), without the variable contribution of HBeAg. Moreover, as mentioned above, the ratio of empty vs. complete virions in different patients varies widely. Among other factors, this ratio may reflect the efficiency of intrahepatic assembly of empty vs. pgRNA-containing capsids, which, together with the efficiency of reverse transcription and virion assembly and secretion, ultimately determine the ratio of empty vs. complete virions in the blood (Figure 1). Thus, measurement of both complete and empty virions in the blood can also help monitor these events in the liver, which could reflect the changing viral activities, host physiology, and virus-host interactions. 

#### 4.1.2. Serum HBV RNA 

As mentioned earlier, there has been a lot of interest recently to use serum HBV RNA as a surrogate to monitor hepatic HBV CCC DNA. In principle, release of RNA-containing virions would be independent of viral DNA synthesis and could reflect expression of pgRNA, its packaging into immature NCs and subsequent envelopment and secretion. Due to the ease and sensitivity in RNA detection and quantification, as compared to HBcAg (empty virions), serum HBV RNA could thus be a convenient marker to monitor intrahepatic CCC DNA, when viral DNA synthesis is effectively suppressed with antiviral therapy [12]. Consistent with this expectation, various reports have shown that serum HBV RNA levels decreased much less and slower than serum HBV DNA during therapy with RT inhibitors [11,13,59,60]. A quick RNA decrease early during therapy with RT inhibition may predict viral clearance [60], and conversely, the persistence of serum HBV RNA after RT inhibition was associated with a risk of viral rebound after discontinuation of treatment [10]. It was also reported that interferon treatment could induce a more significant decrease in serum HBV RNA than RT inhibitors [57,59], presumably due to the inhibitory effect of interferon, but not RT inhibitors, on the levels of HBV RNA in the hepatocytes. 

As discussed above, it is important to more clearly define both the nature of the viral RNA as well as the particles/vesicles that harbor the RNA in the blood, in order to more reliably and accurately interpret the meaning of serum HBV RNA levels in terms of viral gene expression and replication, and response to therapy. If the serum HBV RNA indeed represents immature virions (i.e., enveloped immature NCs containing pgRNA (and RT)), it could reflect hepatic CCC DNA levels. However, the reported detection of truncated HBV RNAs in the serum samples, which was likely transcribed from integrated HBV DNA and might be released to the blood in the absence of any other viral markers, may instead reflect release of non-functional viral RNAs in some sort of vesicles in the absence of either capsid or virion assembly. If this turns out to be true, serum HBV RNA levels obviously would not reflect hepatic CCC DNA level or its transcriptional activity. In addition, therapeutics designed to block pgRNA packaging into NCs [66], currently under active development, would lead to a loss of serum HBV RNA-containing virions despite CCC DNA persistence in the liver. In this case, measurement of serum empty virions (HBcAg), not the putative RNA virions, would help monitor hepatic CCC DNA levels. 

In summary, quantitative and sensitive measurement of serum HBcAg (empty virions), and possibly, HBV RNA, in combination with the classical serum viral markers (HBV DNA, HBsAg, HBeAg), have the potential to provide a more accurate view of hepatic CCC DNA level and its transcriptional activity, and thus help monitor viral persistence and response to antiviral treatment. In particular, the decision of when to stop antiviral therapy remains an important issue in the treatment of chronic hepatitis B [22,95]. It was recently estimated that it may require life-long treatment (average 52 years) with current antiviral therapy, even in the absence of drug resistance, to clear HBsAg [96], the current “gold standard” for a cure of chronic HBV infection. However, this may be an overly pessimistic view, and indeed the use of HBsAg loss may be an overly stringent criterion for a cure - at least for direct acting antivirals, if the remaining serum HBsAg is produced exclusively from integrated HBV DNA following the loss of hepatic CCC DNA and does not have a major pathological consequence. In such a scenario, a significant decline and loss of serum HBcAg may signify CCC DNA clearance (or stable silencing) in the liver, which could help guide the safe discontinuation of therapy. For these individuals, continued treatment to block viral replication or induce CCC DNA loss is unlikely to be beneficial. If a complete loss of HBsAg is still desired in this situation [97], alternative treatment strategies targeted at the integrated HBV DNA would be required. 

### 4.2. Empty Virions as a Candidate for a New Generation of HBV Vaccine?

The empty virions may also form the basis for a new generation of HBV vaccine. As mentioned above, the 1^st^ generation of HBV vaccine was derived directly from plasma of infected patients and would have contained HBsAg particles, empty virions, and complete virions (inactivated). Due to theoretic safety concerns with the use of human plasma, this highly effective vaccine was replaced by the current recombinant (2nd generation) HBV vaccine that contains only one of the HBV envelope protein, S, and no capsid protein. Though it has proven to be safe and effective in most cases, the recombinant S vaccine fails to induce a sufficient response in some vaccinees [98]. Also, as the recombinant vaccine elicits predominantly an antibody response targeted at a single common epitope, the so-called “a” determinant in S, HBV can evolve mutations in this epitope to escape the vaccine-induced antibodies [98,99,100]. To potentially exacerbate the vaccine escape problem further, RT inhibitors, the most widely used treatment for chronic HBV infection, have been shown to select vaccine escape mutants as well as drug-resistant mutants. As alluded to above, due to the overlap of the RT and envelope genes, certain drug resistant mutations in the RT gene also encode vaccine-escape S proteins in the overlapping S gene [79,101,102]. A potential 3rd generation HBV vaccine could be based on empty virions, which would contain all of the viral structural proteins but no genome. In principle, such a vaccine should be as safe as the current generation of vaccine but may help overcome the limitations of the current vaccine by providing additional antigenic determinants (and possibly better mimics of complete virions) for both humoral and cellular immune responses, the latter of which target dominantly the internal capsid protein [98]. Thus, an empty virion-based vaccine may prove to be effective for therapeutic as well as prophylactic purposes.

## 5. Summary

The discovery of the classical Au antigen (HBsAg spheres and filaments) half a century ago proved instrumental in the identification of HBV that predated the discovery of both the complete, infectious virions as well as the viral genetic material. These incomplete, non-infectious, subviral particles also formed the basis for the subsequent development of highly efficacious measures (vaccines and biomarkers) to prevent and manage this deadly viral infection. The more recent identification and characterization of the genome-free (empty) HBV virions, and of potentially RNA-containing HBV virions, challenge long-standing dogmas in the field regarding HBV assembly and present interesting questions about the biological functions of these particles. Further studies on the incomplete HBV particles, as well as the complete virions, should not only deepen our understanding of HBV replication and morphogenesis and but will also likely bring about useful and timely tools (novel markers and vaccine candidates) to facilitate current efforts to develop a cure for HBV infection.
